# Impact on Patient Care of a Multidisciplinary Center Specializing in Colorectal and Pelvic Reconstruction

**DOI:** 10.3389/fsurg.2018.00068

**Published:** 2018-11-19

**Authors:** Alejandra Vilanova-Sánchez, Carlos Albert Reck, Richard J. Wood, Cristina Garcia Mauriño, Alessandra C. Gasior, Robert E. Dyckes, Katherine McCracken, Laura Weaver, Devin R. Halleran, Karen Diefenbach, Dennis Minzler, Rebecca M. Rentea, Christina B. Ching, Venkata Rama Jayanthi, Molly Fuchs, Daniel Dajusta, Geri D. Hewitt, Marc A. Levitt

**Affiliations:** ^1^Pediatric Surgery, Colorectal and Pelvic Surgery Division, Hospital Universitario La Paz, Madrid, Spain; ^2^Center for Colorectal and Pelvic Reconstruction (CCPR), Nationwide Children's Hospital, Columbus, OH, United States; ^3^Vienna General Hospital, Vienna, Austria; ^4^Nationwide Children's Hospital, Columbus, OH, United States

**Keywords:** anorectal malformation (ARM), hirschsprung disease, multidiscipinary collaboration, combined procedure, colorectal surgery, impact on patient care

## Abstract

**Aim of the study:** Many patients with an anorectal malformation (ARM) or pelvic anomaly have associated urologic or gynecologic problems. We hypothesized that our multidisciplinary center, which integrates pediatric colorectal, urologic, gynecologic and GI motility services, could impact a patient's anesthetic exposures and hospital visits.

**Methods:** We tabulated during 2015 anesthetic/surgical events, endotracheal intubations, and clinic/hospital visits for all patients having a combined procedure.

**Main results:** Eighty two patients underwent 132 combined procedures (Table 1). The median age at intervention was 3 years [0.2-17], and length of follow up was 25 months [7-31]. The number of procedures in patients who underwent combined surgery was lower as compared to if they had been done independently [1(1-5) vs. 3(2-7) (*p* < 0.001)]. Intubations were also lower [1[1-3] vs. 2[1-6]; *p* < 0.001]. Hospital length of stay was significantly lower for the combined procedures vs. the theoretical individual procedures [8 days [3-20] vs. 10 days [4-16]] *p* < 0.05. Post-operative clinic visits were fewer when combined visits were coordinated as compared to the theoretical individual clinic visits (urology, gynecology, and colorectal) [1[1-4] vs. 2[1-6]; *p* = < 0.001].

**Conclusions:** Patients with anorectal and pelvic malformations are likely to have many medical or surgical interventions during their lifetime. A multidisciplinary approach can reduce surgical interventions, anesthetic procedures, endotracheal intubations, and hospital/outpatient visits.

## Introduction

Anorectal malformations (ARM), and a variety of complex pelvic anomalies have a high prevalence of urologic and gynecologic malformations with future consequences in urologic and fecal continence as well as sexual and reproductive health (1–4). Also, patients with spinal anomalies such as myelomeningocele have a high prevalence of neurogenic bladder and bowel leading to incontinence (5). Due to the complexity of these malformations these patients require multiple clinic visits, hospital admissions, and surgical procedures during their lifetime. Moreover, due to the broad scope of these malformations, numerous specialists in different surgical areas such as pediatric colorectal surgery, urology, and gynecology need to be involved in their care.

Good outcomes have been well described with surgical and medical multidisciplinary teams, reducing morbidity as well as cost and hospital stays when treating complex medical conditions, in both adult and pediatric populations (6–8). However, to our best knowledge, there is no reference showing the impact of a multidisciplinary approach in a Pediatric Colorectal Center.

In 2014, we developed a Center devoted to complex colorectal and pelvic care, with the primary goal to reduce the social and familial impact, the surgical and medical burden of care in this particular population. We aimed to describe our experience in a multidisciplinary center that treats patients with complex pelvic malformation and assess the impact of the collaborative approach to their care regarding surgical procedures, anesthetics events, and hospital and clinic visits.

## Methods

### Data collection

A retrospective review of patients having multidisciplinary treatment, with the involvement of more than one surgical team was performed from 1/2015 to 1/2016. The type of malformation, teams involved, type of anesthesia, procedures performed, length of hospital stay, number of clinic visits, complications, and duration of follow up were recorded.

We compared our results with the data we would have obtained if the procedures would have been done without the collaboration between services, meaning that a patient would have had individually procedures with individual anesthetic events, endotracheal intubation (if required), and single hospital admission and post-operative clinic visits.

To calculate the theoretical length hospital stay for individual procedures, the mean stay for the individual surgeries performed in our service during the same period for the same group of surgeons (year 2015) was recovered from the hospital database. For example: Mean of Hospital stay of Bilateral reimplant: 2 days. Mean of Colostomy closure: 4 days.

If they would had the procedures separately, they would have needed 6 days in 2 different hospital admission, 2 different anesthetics events and endotracheal intubations, 2 separate postoperative clinic visits and therefore hospital visits. If with the collaborative approach we found a patient having a colostomy closure and bilateral reimplant in the same anesthetic event with the same endotracheal intubation who needed 5 days of hospital admission we calculated: 1 surgical/anesthetic event, 1 endotracheal intubation, and only 1 post-operative clinic visit. That patient would have saved 1 anesthesia, 1 endotracheal intubation and 1 day of hospital admission.

### Statistical analysis

Age of patients at time of combined surgical intervention, combined procedures per patient, anesthetic events, number of endotracheal intubation, hospital stay and clinic visits with and without collaboration were compared using Mann—Whitney test for continuous variables and chi-square test for categorical variables, and data presented as median (range) or frequencies (percentages) accordingly. Analyses were conducted GraphPad Prism v.6 (GraphPad Software; La Jolla, CA), with a two-sided *p*-value of < 0.05 considered statistically significant.

## Selection of patients for combined procedures

All patients able to have a combined procedure were discussed in a collaborative meeting. In that weekly team meeting, all the care providers in our Center are present: pediatric colorectal surgeons, pediatric urologists, pediatric and adolescent gynecologists, nurses and nurse practitioners, anesthesiologists, operating room staff, and social workers. We discuss the type of intervention needed, anesthetics risk, and modality of postoperative treatment and pain control. We also discussed the post-operative follow up plan for all of the disciplines involved in their care.

## Limitation of the study

The theoretical number of independent procedures, independent anesthetic procedures, endotracheal intubations, and independent hospital admissions days are data that were telescope and attributed to our population. Due to the variables that we are comparing we could not compare the same procedures done combined and separately for the same population of patients during the same period. The most objective way to compare both approaches was to compare medians assuming the patients have the same characteristics and demographic characteristics in both groups. Regarding complications, we could not compare the rates of complications for the procedures done separately and combined as we did not have the data for the independent procedures available. Starting in 2016, the data has been collected prospectively (for both groups), and we will be able to report complications rate comparing both groups in the future.

## Results

During the 1 year study period, a total of 285 patients underwent 469 surgical procedures in our Center. Of these, 82 patients underwent 132 combined procedures including: urology, gynecology plus colorectal (87), urology plus colorectal (34), or gynecology plus colorectal (11). Of the 82 patients, 56 (68%) were previously operated elsewhere and attended our clinic for definitive pelvic reconstruction or evaluation for a reoperation. Twenty six patients (32%) had not undergone prior definitive surgery before evaluation or treatment at our Center. The median age at the time of the combined procedures was 3 years [0.2–21]. The most frequent type of malformation was complex ARM, followed by cloaca and spinal anomalies (Table 1).

**Table 1:** Description of the services involved in the combined procedures.

Malformation	Complex ARM	75
	Spinal Anomaly	5
	Sacrococcygeal Teratoma	2
Combined procedures	Colorectal + Urology + Gynecology	87
	Colorectal + Urology	34
	Colorectal + Gynecology	11
Reason for collaboration	Practical Advantage	115
	Tissue sharing	9
	Single Pelvic Exploration	8

We divided the combined procedures into 3 categories based on the main reason for the collaboration (Table 2).

**Table 2:** Description of the combined porcedures and reason for combined surgery.

**Type of collaboration**	**Number of procedures**	**Combined procedures**
Practical advantage	115	75 Cystoscopy + Examination under anesthesia + vaginoscopy 8 Colostomy closure + assessment Mullerian structures 6 Colostomy closure + orchidopexy 5 Colostomy closure + circumcision 4 Colostomy closure + hypospadias repair 3 Colostomy closure + ureteral reimplantation 2 Colostomy closure + scrotoplasty 1 Colostomy closure + malone + ureteral reimplant + vesicostomy closure 1 Colostomy closure + nephrectomy 1 Colostomy closure + orchiectomy + scrotoplasty + utricule resection 1 Redo PSARP + introitoplasty + vaginal septum removal 1 Redo PSARP + lap Malone + resection remnant of the original fístula (ROOF) + penoscrotal web release 1 Redo PSARP + lap Malone + circumcision + penoscrotal transposition 1 Redo PSARP + Malone + orchidopexy + scrotoplasty 1 Redo PSARP + ROOF excision + ureteral reimplant 1 Sigmoid resection + Malone + Mitrofanoff revision +Inspection mullerian structures + vaginoscopy 1 Neo-Malone + Mitrofanoff revision + cystoscopy
Tissue sharing	9	5 Sigmoid colon used for bladder augment +BN sling + sigmoid resection in dysfunctional colon + Mitrofanoff 3 Split Appendix used for Malone + Mitrofanoff 1 Redo PSARP + rectal patch used for urethral stricture repair
Single pelvic exploration	8	2 Colonic pull through + bladder augment + BN sling + Mitrofanoff + vaginal replacement + ileostomy creation 1 Redo Cloaca repair + vaginal replacement + bladder augment 1 Redo Cloaca repair + ileostomy creation + left ureteral reimplant + vaginal replacement 1 Redo Cloaca repair + Neo-Malone + ileostomy creation + bilateral ureteral reimplant + mitrofanoff + vaginal Replacement 1 Redo PSARP + Bladder augmen t+ Monti + Introitoplasty +inspection mullerian structures 1 Cloaca repair + Bladder augment + vesicostomy + nephrectomy + vaginal replacement
Total	132

*PSARP, Posterior sagittal anorectoplasty; ROOF, Remamnt of the origianl fistula; BN, Bladder neck*.

The first group of procedures were those we decided to be combined in order to save an anesthetic, visits to the hospital, and/or for a practical advantage (115). Seventy five of these procedures (57%) were minor procedures such an examination of the perineum under anesthesia combined with vaginoscopy and cystoscopy, with the three teams present at the procedure. This simple combination of procedures saved 150 anesthesia inductions, 150 hospital visits and allowed operative planning for future reconstruction if needed.

The second group of combined procedures were those which could not have been done without collaboration due to sharing of tissues (9) (Table 2). These include 3 appendices used for both Malone and Mitrofanoff, 5 sigmoid colons used for both bladder augment and sigmoid resection (in patients with colonic dysmotility needing a colonic resection) and 1 redo PSARP and rectal patch used for urethral repair of urethral stricture after primary PSARP.

The third group of patients was selected for a combined procedure to enter the pelvis only once in complex pelvic malformations, including the 3 combined teams working together performing complex urologic, gynecologic and colorectal reconstruction (8).

The median number anesthetic events per patient and therefore hospital visits were significantly lower in those who underwent combined procedures vs. if the procedures would have been done independently [combined 1 [1-5], individual 3[2-7]].

The number of patients who required endotracheal intubation in any of their combined procedures was 36, with a total of 42 endotracheal intubations performed. The median number of intubations per patient was significantly lower when combined procedures were performed vs. theoretical individually performed surgeries. [combined; 1[1-3], individual 2[1-6]; *p* < 0.001] (Table 3).

**Table 3:** Comparison of the number of anesthetic inductions, clinic vistis, and endotracheal intubations perfomed per patient with and without a multidisciplinary approach.

**Intervention**	**With multidisciplinary team**	**Without multidisciplinary team**	**Total interventions saved**	**Intervention saved per patient**	***p*-value**
Anesthetic inductions	132	346	214	2.6 (1–6)	*p* < 0.001
Endotracheal intubations	50	101	51	0.6 (0–3)	*p* < 0.001
Clinic visits	67	156	89	11.17 (1–2)	*p* < 0.001

A total of 43 procedures required hospital admission. The median number of hospital stay in days was significantly lower for the combined procedures vs. theoretical individual procedures (8 days [3-20] vs. 10 days [4-16]; *p* < 0.001).

A total of 46 patients had a total of 67 multidisciplinary clinic visits. The median number of clinic visits and therefore visits to the hospital during the study period per patient was significantly lower when combined clinic visits were coordinated vs. theoretical individually visits (urology,gynecology, and colorectal) [combined; 1[1-4], individual 2[1-6]; *P* = < 0.001].

The median length of follow up has been 25 months [7-31].

In the post-operative period, 6 patients had complications that required a conservative treatment (5 wound infections, 1 scrotal hematoma). Three patients needed reintervention in the same hospital admission (2 pelvic abscesses with IR drainage placement, 1 rectovaginal fistula with colostomy creation). In the long term follow up, 1 patient needed a Mitrofanoff revision, and 1 malone revision, both for a skin level stricture.

## Discussion

Patients with congenital anorectal and pelvic malformations as well as other many chronic health impairments require regular episodes of hospital visits or anesthetic exposures. These children are medically fragile and need frequent hospitalizations, emergency department visits, and surgical procedures. The presence of a chronic condition, such as complex pelvic malformation, may also limit or alter social interactions, due to fecal or urinary incontinence, and distinguish children from their peers, which can increase their anxiety and depression in early ages (9). It has been well documented in many medical fields, in both adults and children, that a multidisciplinary approach can improve healthcare in complex illnesses. With a multi-specialist work team approach, health care burden, patient self-perception of illness and family responsibilities can be minimized as well as a reduction of surgical procedures and therefore exposure to anesthetics (10–14). Although, this has been proven in other conditions, this is the first study to our knowledge reporting on the impact on medical care associated with an interdisciplinary approach for children with complex congenital pelvic/anorectal malformations.

In this study, we analyzed the burden of care in patients presenting to our multidisciplinary Colorectal and Pelvic Reconstruction Center who underwent a surgical procedure from January 2015 and January 2016. We provided combined procedures with more than one service involved to 82 patients, performing 132 combined procedures. The majority of combined procedures 75 (57%) were done with the primary objective of minimizing the hospital visits, or to shorten anesthetics exposure or minor procedures. This first group of combined procedures saved 150 anesthesia inductions and 150 hospital visits.

Our goal was to minimize multiple anesthetic exposures in children, which have been related to an increased risk for later development of ADHD and mental disabilities (15, 16). In studies demonstrating an association of anesthesia with disability, only children with multiple anesthetic exposures have been associated with deficits, but an effect with a single exposure has not been identified (17, 18). Environmental factors though may be important in the development of mental issues after anesthesia (19).

We also reduced the number of endotracheal intubations by 50% (from 101 to 50) with a mean saved per patient ratio of 0.6 (range 0–3) (*p* < 0.0001). This reduction directly minimized the risk of developing congenital subglottic stenosis in children due in part to multiple endotracheal intubations or long period of intubations (20–22).

The second groups of procedures were scheduled as combined cases for tissue sharing advantages. We used the appendix for both Malone and Mitrofanoff in children with neurogenic bowel and bladder (23). This way we avoided the risk of a new intervention or the risk of the creation of either a neo-Malone or a small bowel Monti procedure with a bowel anastomosis. We also shared the sigmoid in 5 children needing colonic resection for colonic dysmotility and needing a bladder augmentation. In a patient with an anal and urethral stricture after a primary PSARP, we used a rectal patch for repair of a post-operative urethral stricture done at the time of the redo PSARP. These procedures could not have been done without a collaborative approach.

The third groups of patients are those with very complex urologic, gynecologic, and colorectal diseases requiring repair of all three organ systems. We had several reasons to combine their operations in addition to saving hospital visits, anesthetic exposure and the number of endotracheal intubations namely the ability to avoid entering the pelvis multiple times. The distinct advantages to this approach include: Colorectal surgery involving colonic pull through and pelvic anastomosis have been directly related with infertility (24–26), and multiple pelvic and abdominal surgeries are directly associated with a higher risk of bowel obstruction and the need for emergent surgery. Adhesiolysis in repeat abdominal surgery may increase the incidence of inadvertent bowel injury and increases the operating time and as well as future chronic abdominal and pelvic pain (27, 28).

The total hospital days saved was 103 and the mean saved per patient was 1.25 (range 0–5) (*p* < 0.0001). By reducing the number of days of hospital admission, we reduced the burden of medication, antibiotics, the risk of nosocomial infection and psychologic and economic impact on the patient and the family. By lowering the post-operative clinic visits, we also reduced the cost of travel to the clinic for the family as well as the number of unattended work/school days. Post-operative combined clinic visits were reduced by 42%, and every patient saved at least 1–2 clinic visits.

In summary, we can conclude that patients with anorectal and pelvic malformations are likely to have many medical or surgical interventions during their lifetime. Our data shows that if they are treated using a multidisciplinary approach, hospital length of stay, clinic visits and surgical and anesthetic procedures can be minimized. This reduces both the burden of care as well as the financial impact of medical care. We believe that this model of care could be reproducible in other parts of the health care system.

## Author's note

This study was reviewed and approved by Institutional Review Board at Nationwide Children's Hospital, Columbus Ohio, with the study registry number IRB14-00232 being part of the Colorectal registry at the Center for colorectal and pelvic reconstruction, Nationwide Children'sHospital.

## Author contributions

AV-S: Main author. CR, AG, KM, RD, DM, KD, CC, and DD: Data collection. RW, VJ, GH, DRH, and MF: Editor of manuscript. CG, RR, and LW: Data analysis. ML: Senior author. All authors of this research paper have directly participated in the planning, execution, or analysis of this study. All authors of this paper have read and approved the final version submitted.

### Conflict of interest statement

The authors declare that the research was conducted in the absence of any commercial or financial relationships that could be construed as a potential conflict of interest.
